# Optimal Design of an Ultrasmall SOI-Based 1 × 8 Flat-Top AWG by Using an MMI

**DOI:** 10.1155/2013/636912

**Published:** 2013-07-31

**Authors:** Hongqiang Li, Yaoting Bai, Xiaye Dong, Enbang Li, Yang Li, Yu Liu, Wenqian Zhou

**Affiliations:** ^1^School of Electronics and Information Engineering, Tianjin Polytechnic University, No. 399 Binshuixi Road, Xiqing District, Tianjin 300387, China; ^2^School of Physics, Faculty of Engineering, University of Wollongong, Wollongong, NSW 2522, Australia

## Abstract

Four methods based on a multimode interference (MMI) structure are optimally designed to flatten the spectral response of silicon-on-insulator- (SOI-) based arrayed-waveguide grating (AWG) applied in a demodulation integration microsystem. In the design for each method, SOI is selected as the material, the beam propagation method is used, and the performances (including the 3 dB passband width, the crosstalk, and the insertion loss) of the flat-top AWG are studied. Moreover, the output spectrum responses of AWGs with or without a flattened structure are compared. The results show that low insertion loss, crosstalk, and a flat and efficient spectral response are simultaneously achieved for each kind of structure. By comparing the four designs, the design that combines a tapered MMI with tapered input/output waveguides, which has not been previously reported, was shown to yield better results than others. The optimized design reduced crosstalk to approximately −21.9 dB and had an insertion loss of −4.36 dB and a 3 dB passband width, that is, approximately 65% of the channel spacing.

## 1. Introduction

**Figure 1:**
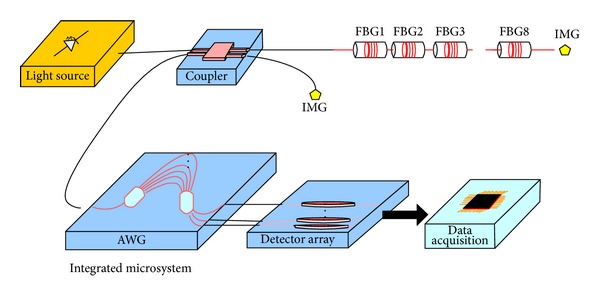
FBG demodulation system based on AWG.

A wearable optical fiber-grating- (FBG-) based sensor in intelligent clothing for human body temperature measurement has previously been demonstrated [1]. Likewise, we designed an arrayed-waveguide grating (AWG) demodulation integration microsystem (Figure 1) with a new kind of FBG demodulation scheme, and an ultrasmall silicon-on-insulator- (SOI-) based 1 × 8 normal AWG without flat spectral response [2], which is a key component in demodulation microsystems, is designed.

Based on spectral response, an AWG can be classified as a Gaussian type or a flat-top type. The application of Gaussian-type AWGs is limited by the influence of external factors because of their small 3 dB bandwidth. By contrast, flat-top type devices have numerous advantages, such as the facilitation of high-speed modulation and insensitivity to center wavelength shift. A large number of methods have been introduced to achieve a flattened AWG spectral response [3–7] in silica- or InP-based AWGs, but rarely demonstrated in SOI-based AWGs, where the very high contrast makes the design of such MMI couplers and AWGs difficult. These methods have a number of limitations, such as an increase in the size or the insertion loss of the device. Among these methods, the connection of an MMI section to the end of the input waveguide of an AWG is relatively simple and effective. In 2003, Dai introduced a method wherein a tapered MMI, which was connected to the end of the input waveguide of the AWG, was used to flatten the passband of an AWG. Insertion loss and crosstalk were −2.35 dB and −35.6 dB, respectively. The 3 dB passband width was approximately 80% of the channel spacing [8]. In 2005, Khan reported on the passband broadening of an AWG demultiplexer on Si/SiO_2_ with a tapered output waveguide and an MMI coupler connected to the end of a tapered input waveguide. The crosstalk level was below −30 dB. The insertion loss was approximately 5.6 dB for the central channel. The 3 dB passband width was approximately 42% of the channel spacing [9]. In 2006, An et al. designed a flat-response silica-based AWG with 40 channels using a symmetrical MMI attached to the end of an input waveguide. The insertion loss ranged from 5.2 dB to 7.5 dB, and the crosstalk was less than −20 dB [10]. In 2012, Pathak et al. reported on an ultrasmall AWG on SOI with flattened spectral response that employs an MMI aperture with insertion loss of −3.29 dB and crosstalk of −17 dB [11].

In this paper, we design four optimized MMI-based methods to achieve a flattened spectral response of compact silicon AWGs used in an AWG demodulation integration microsystem. SOI is selected as the material during design to produce compact silicon AWGs [12]. The flattened AWGs are simulated using the beam propagation method (BPM). For each method, performance parameters such as insertion loss, crosstalk, and 3 dB bandwidth are obtained. We then analyzed and compared the four methods.

## 2. Design and Optimization

### 2.1. Use of a Symmetrical MMI to Flatten the Spectral Response

**Figure 2:**
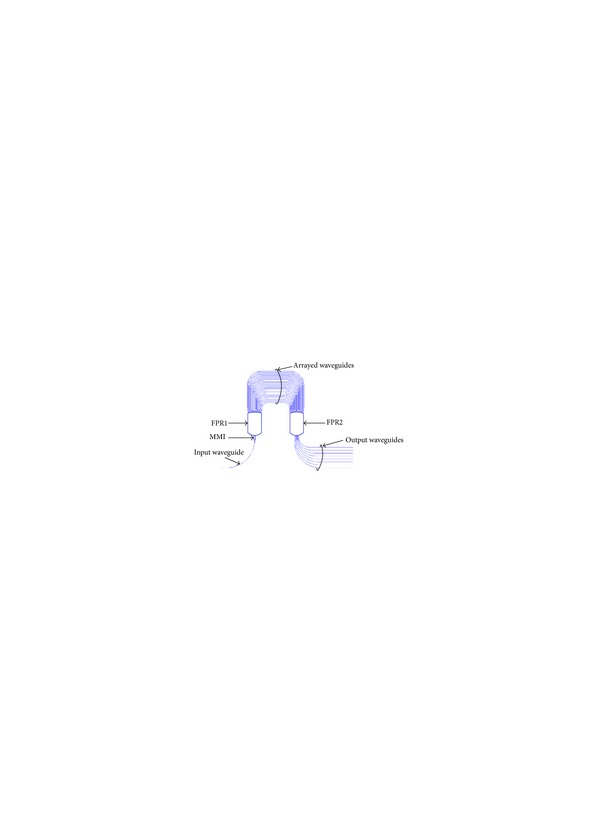
Schematic diagram of an MMI-AWG.

The structure of an MMI-AWG demultiplexer is shown in Figure 2. An AWG comprises an input waveguide, numerous output waveguides, two free propagation regions (FPRs), and arrayed waveguides. To broaden the spectral response of an AWG demultiplexer, a symmetrical MMI coupler (with length *L*_MMI_) is connected to the end of the input waveguide. The input light is radiated to the first FPR to excite the arrayed waveguides. After traveling through the arrayed waveguides, the light beam constructively interferes at one focal point in the second FPR. The location of the focal point depends on the wavelength.

According to the self-image theory, a twofold image can be formed in the MMI output surface region. For MMI imaging, the effective width for each order mode is approximately equal to that for the fundamental mode. The effective width *W*_*e*_ is given by

(1) $$\begin{array}{c} W_{e}=W+\frac{\lambda_{0}}{\pi }\cdot {\left(\frac{n_{c}}{n_{r}}\right)}^{2\sigma }{\left(n_{r}^{2}-n_{c}^{2}\right)}^{-1/2}, \end{array}$$

where *W* is the width of the MMI section, *λ*_0_ is the wavelength in vacuum, *σ* = 0 (for the TE case) or 1 (for the TM case), and *n*_*r*_ and *n*_*c*_ are the refractive indexes for the core and the cladding, respectively. For a high refractive index difference, *W*_*e*_ ≈ *W*. When

(2) $$\begin{array}{c} L_{\text{MMI}}=\frac{1}{N}\cdot \frac{3}{4}\cdot L_{\pi }, \end{array}$$

an *N*-fold image will be produced, where *L*_*π*_ is given by

(3) $$\begin{array}{c} L_{\pi }\approx \frac{4n_{r}W_{e}^{2}}{3\lambda_{0}}. \end{array}$$

Thus, the twofold image is formed at the end of the MMI section with the following length:

(4) $$\begin{array}{c} L_{\text{MMI}}=\frac{3}{8}\cdot L_{\pi }. \end{array}$$

**Figure 3:**
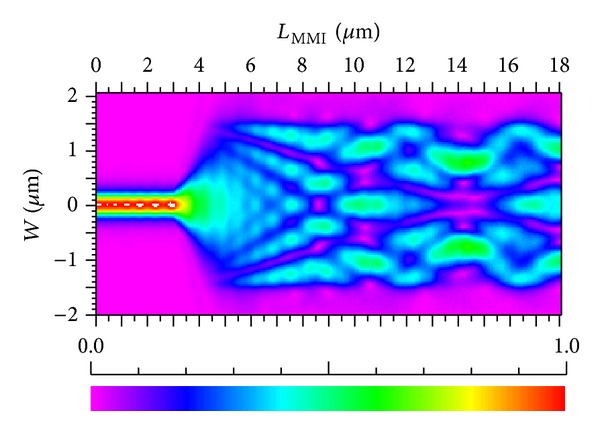
Optical field distribution of an MMI.

The given formula can be used to determine the values of the major parameters *W* and *L*_MMI_ for the design of the MMI. First, based on the requirements of 3 dB bandwidth, *W* is set to 3 *μ*m. Then, according to formula (4), *L*_MMI_ is found to be approximately 10.05 *μ*m. MMI is simulated by using the BPM method, and optical field distribution is shown in Figure 3. From the figure, distinct image points are evident.

**Figure 4:**
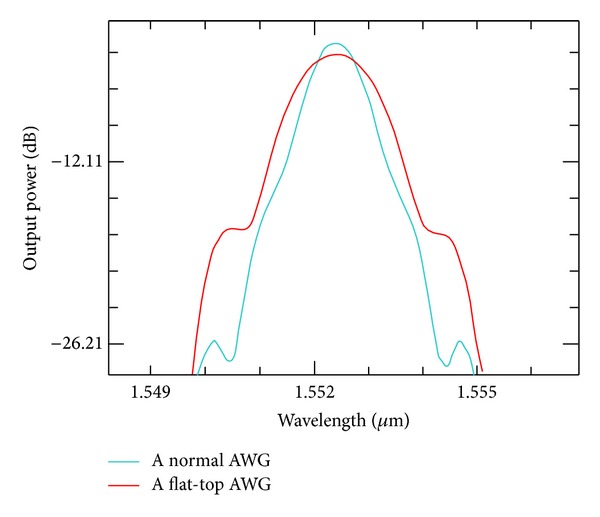
Contrast of single-channel output spectrum.

Two 1 × 8 AWGs, a normal AWG and an MMI-AWG, both having the same structure except for the input region, are designed and calculated. The widths and heights of the AWG waveguides are 0.35 and 0.22 *μ*m, respectively. The other AWG parameters are given in Table 1. Based on the simulation and analysis of AWGs, the optimized size of the MMI is set to 3 *μ*m × 13 *μ*m. The single-channel output spectrum contrast of the same AWG device with and without MMI is shown in Figure 4. The result shows that the passband is flattened to 1.28 nm at 3 dB. The insertion loss and crosstalk are increased to −5.76 and −19.54 dB, respectively.

### 2.2. Use of a Tapered MMI to Flatten the Spectral Response

**Figure 5:**
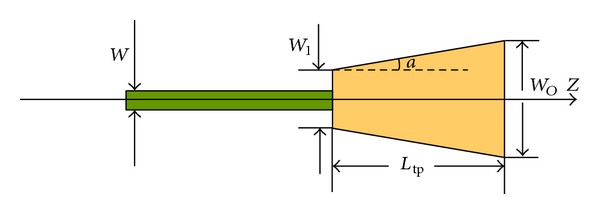
Geometrical configuration of a tapered MMI.

To increase the design degrees of freedom to obtain a superior flattened spectrum, a tapered MMI (Figure 5) is used to flatten the passband of an AWG.

The width of a tapered MMI is given by

(5) $$\begin{array}{c} W\left(z\right)=W_{O}+\left(W_{I}-W_{O}\right)\left(1-\frac{z}{L_{\text{tp}}}\right), \end{array}$$

where *z* is along the propagation direction, *L*_tp_ is the length of the tapered MMI, and *W*_*I*_ and *W*_*O*_ are the entrance and exit widths of the tapered MMI, respectively, (Figure 5). Thus, *L*_*π*_ yields the following expression:

(6) $$\begin{array}{c} L_{\pi }\approx \frac{4n_{r}{\left(W_{O}\cdot W_{I}\right)}^{2}}{3\lambda_{0}}. \end{array}$$

Considering that the separation between the two image peaks is mainly determined by *W*_*O*,*I*_ and *L*_tp_ are expressed using *W*_*O*_ and tapering angle *a*. The following expressions are thus derived:

(7) $$\begin{array}{c} L_{\text{tp}}=\frac{n_{r}\cdot W_{O}^{2}}{2\cdot \left(\lambda_{0}+n_{r}\cdot W_{O}\cdot \tan a\right)},\\ W_{I}=\frac{\lambda_{0}\cdot W_{O}}{n_{r}\cdot W_{O}\tan a+\lambda_{0}}. \end{array}$$

**Figure 6:**
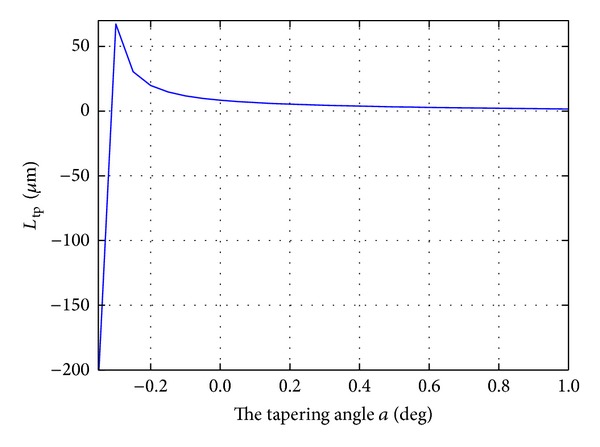
The length *L*_tp_ of the tapered MMI as the tapering angle *a* varies.

In the proposed design, the AWG parameters are the same as those shown in Table 1. According to the 3 dB bandwidth requirements, the exit width *W*_*O*_ is fixed at 3.2 *μ*m. Then the width of the whole structure is then determined by the tapering angle *a* (7). *L*_tp_ is plotted as the tapering angle *a* varies (Figure 6). Figure 6 shows that the length *L*_tp_ can be significantly reduced as the tapering angle *a* increases. In the following section, we analyze how the tapering angle *a* affects the performance (including the crosstalk and insertion loss) of a flat-top AWG.

**Figure 7:**
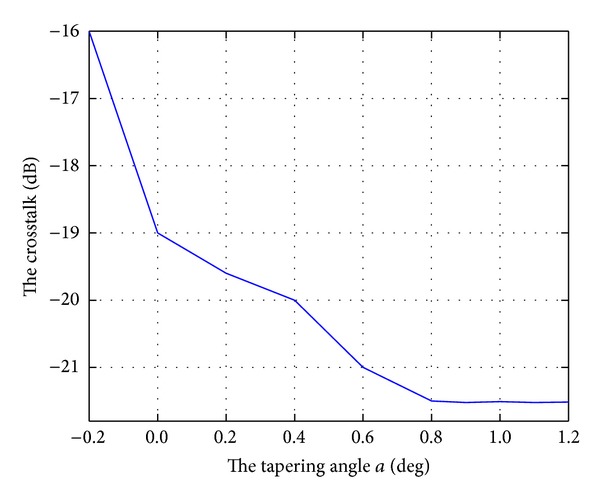
Crosstalk as the tapering angle *a* varies.

Crosstalk is a key parameter for a dense wavelength division multiplexing device. For a practical device, a low crosstalk level must be ensured. For a flat-top AWG, crosstalk increases as the spectral response broadens. Thus, the crosstalk for a flat-top AWG is usually higher than that for a Gaussian-type AWG.  Figure 7 shows the crosstalk as the tapering angle *a* varies. When the tapering angle increases from −0.2° to 0.9°, the crosstalk decreases from approximately −16 dB to −21.7 dB.

**Figure 8:**
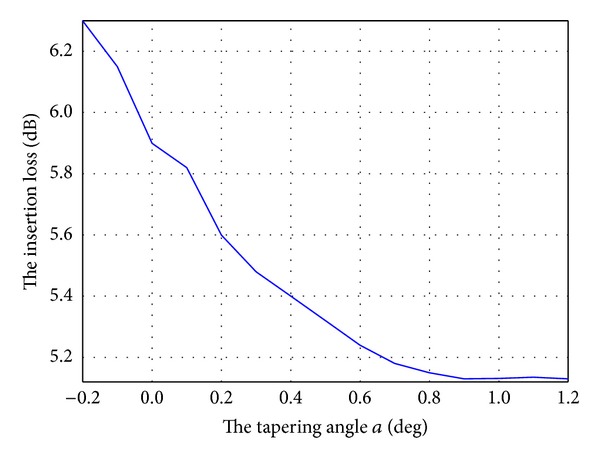
Insertion loss as the tapering angle *a* varies.

Low insertion loss is also important for a practical device.  Figure 8 shows the insertion loss at the central wavelength as the tapering angle *a* varies. Compared with the case of a conventional MMI (*a* = 0°), the insertion loss is reduced by approximately 1 dB when the tapering angle increases to 0.9°.

**Figure 9:**
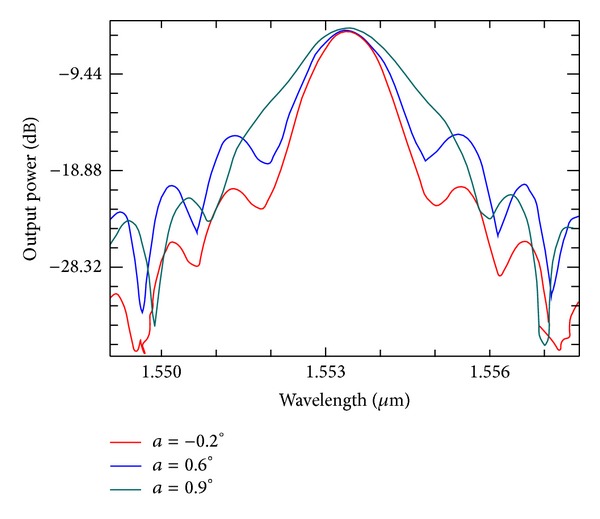
Spectral responses when the tapering angle *a* = −0.2°, 0.6°, 0.9°.

**Figure 10:**
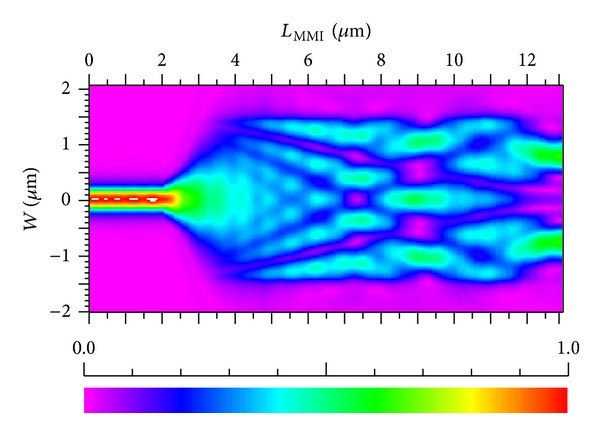
Optical field distribution of a tapered MMI (*a* = −0.9°).

Figure 9 gives the spectral responses when the tapering angle *a* = −0.2°, 0.6°, and 0.9°. This figure shows that the spectral response can be improved by adjusting the tapering angle *a*. When the tapering angle *a* reaches 0.9°, the spectral response is significantly improved compared with the other cases. Moreover, transition is sharpened, crosstalk is reduced to approximately −21.4 dB, insertion loss is −5.11 dB, and the 3 dB passband width is flattened to 1.3 nm. Finally, the device is structured with *W*_*I*_ of 2.88 *μ*m and *L*_tp_ of 10.27 *μ*m. Optical field distribution of the tapered MMI is shown in Figure 10. From the figure, distinct image points are evident. In conclusion, the structure parameters of tapered MMI are advisable.

### 2.3. Use of a Combination of a Tapered MMI and Tapered Input/Output Waveguides to Flatten the Spectral Response

**Figure 11:**
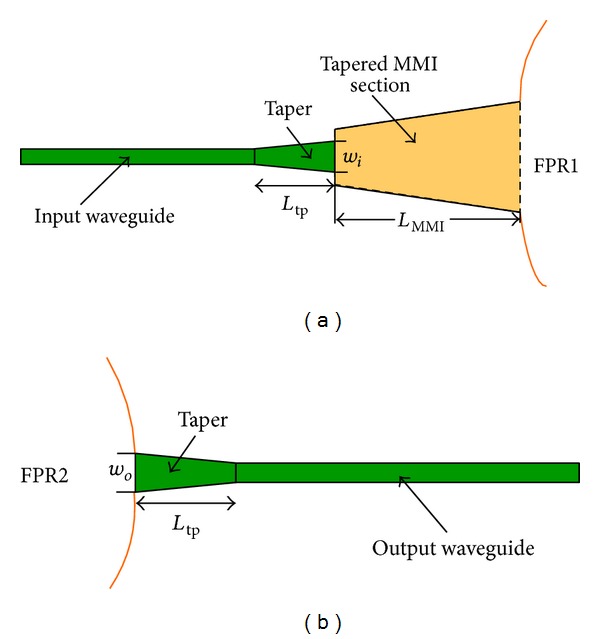
Structure used to flatten spectral responses: (a) section between the input waveguide and FPR1 and (b) section between the output waveguide and FPR2.

This section presents a new kind of structure that can flatten the spectrum while ensuring good planarization with low insertion loss and crosstalk. The improvements based on the structure presented in Section 2.2 include a tapered waveguide inserted before the tapered MMI, such that a preexpanded structure and a tapered waveguide are connected to the output waveguide inlet end.  Figure 11 shows that *w*_*i*_ and *L*_tp_ are the exit width and length of the input tapered waveguide, respectively, whereas *w*_*o*_ is the entrance width of the output tapered waveguide.

**Figure 12:**
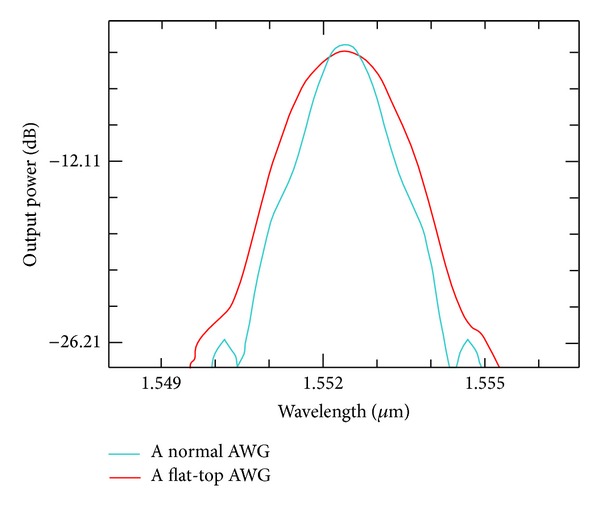
Comparison of single-channel output spectra of flattened and unflattened AWGs.

In the proposed design, the parameters of the ready-to-use AWG are the same as those listed in Table 1. The structure of the tapered MMI is similar to that designed in Section 2.2. Considering that the interval of the AWG output waveguide is 1 *μ*m, *w*_*o*_ = *w*_*i*_ = 0.75 *μ*m is selected. To ensure minimal tapering and conversion of the loss of mold spots, *L*_tp_ is optimally designed at 13 *μ*m. A comparison of the single-channel output spectra of flattened and unflattened AWGs is shown in Figure 12. The result shows that the passband is flattened to 1.31 nm at 3 dB. The insertion loss is decreased to −4.36 dB, and the crosstalk is approximately −21.9 dB.

### 2.4. Use of a Combination of a Symmetrical MMI and Tapered Input/Output Waveguides to Flatten the Spectral Response

**Figure 13:**
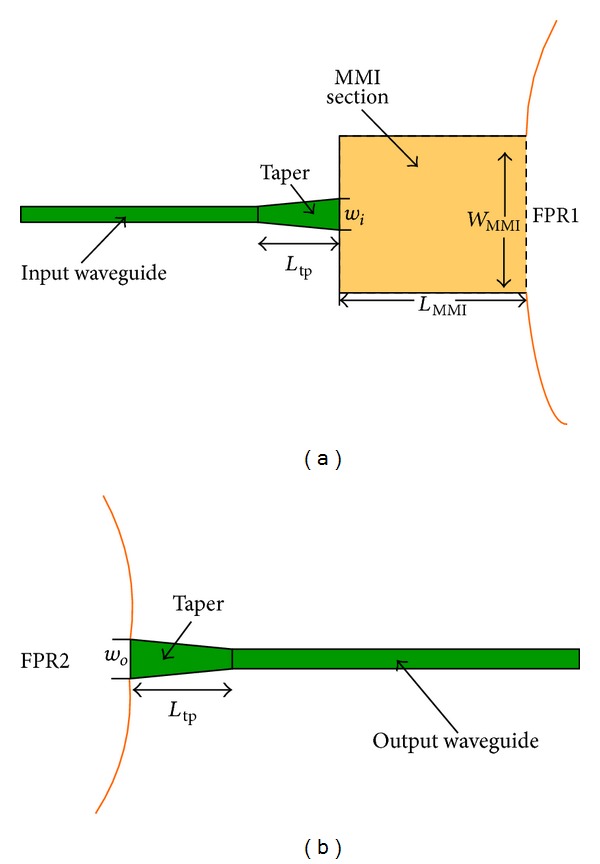
Structure used to flatten spectral responses: (a) section between the input waveguide and FPR1 and (b) section between the output waveguide and FPR2.

Similar to the improvements on the design discussed in Section 2.3, a tapered waveguide is inserted before the MMI and is then connected to the output waveguide inlet end (Figure 13).

**Figure 14:**
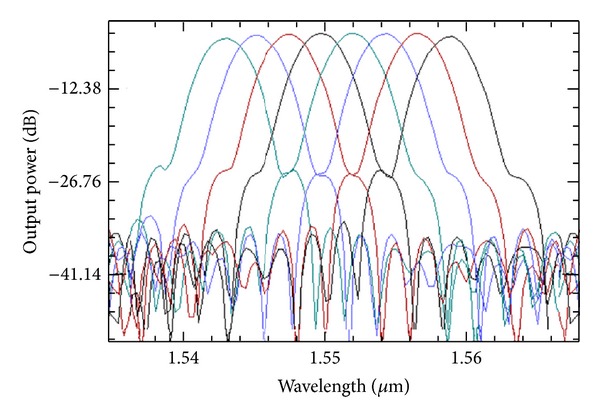
Transmission spectra simulation of a flat-top AWG.

The parameters of the sample AWG are similar to those listed in Table 1. The design process is similar to that reported in Section 2.3. Based on the calculation by using BPM and the optimized design parameters given by (1) and (4), we derive the results shown in Figure 14. The 3 dB passband is 1.3 nm at 65% of channel spacing, insertion loss is −4.66 dB, and crosstalk is approximately −21.5 dB.

## 3. Conclusion

In this paper, the design processes of four kinds of methods used to flatten the spectral response are presented. Through a calculation and simulation conducted by using BPM, the structure is found to be optimally designed, and the 3 dB passband is broadened effectively from 40% to more than 60% of channel spacing for each method.  Table 2 shows a comparison of the four methods. By comparing the four designs, the one reported in Section 2.3 is found to obtain better results than the others, achieving a crosstalk reduction to approximately −21.9 dB, insertion loss of −4.36 dB, and the 3 dB passband width of approximately 1.31 nm, which is 65.5% of channel spacing. Moreover, the design in Section 2.3 has better crosstalk characteristic, simpler, and smaller device structure but little worse insertion loss than the performance reported in [11]. Generally speaking, the designed structures are generally highly compact and exhibit low loss and a wide bandwidth. The designs can thus meet optoelectronic integration requirements.

## Figures and Tables

**Table 1:** Parameter optimization results of the SOI-based AWG.

Parameters	Parameters value
Wavelength	1550.918 nm
Number of output waveguides	8
Number of arrayed waveguides	30
Path length difference	19.76 *μ*m
Length of free propagation region	67 *μ*m
Diffraction order	40
Wavelength spacing	2 nm

**Table 2:** Comparison of the four methods.

Methods to flatten spectral response	Crosstalk (dB)	Insertion loss (dB)	3 dB passband width (nm)
Using a symmetrical MMI	−19.54	−5.76	1.28
Using a tapered MMI	−21.4	−5.11	1.3
Using combination of a tapered MMI and input/output tapered waveguide	−21.9	−4.36	1.31
Using combination of a symmetrical MMI and input/output tapered waveguide	−21.5	−4.66	1.3
